# Specific induction of pp125 focal adhesion kinase in human breast cancer

**DOI:** 10.1038/sj.bjc.6602744

**Published:** 2005-08-23

**Authors:** D O Watermann, B Gabriel, M Jäger, M Orlowska-Volk, A Hasenburg, A zur Hausen, G Gitsch, E Stickeler

**Affiliations:** 1 Department of Obstetrics and Gynecology, University of Freiburg, Hugstetterstr. 55, 79106 Freiburg, Germany; 2 Department of Pathology, University of Freiburg, 79106 Freiburg, Germany

**Keywords:** FAK, matched pairs, breast cancer, specific induction

## Abstract

The pp125 focal adhesion kinase (FAK) is involved in integrin-mediated cell signalling and overexpressed in a variety of solid tumours. Focal adhesion kinase expression has been correlated to invasion and metastasis, but the data on breast cancer are inconclusive. We analysed FAK mRNA, protein levels and expression patterns in primary breast cancer and normal breast tissue. FAK expression on the functional protein level and mRNA was determined in 55 matched pairs of breast cancer and corresponding normal tissue by Western blot, immunohistochemistry and RT–PCR. Using a score ranging from 0 to +5 for Western blots, we determined in normal breast tissue a score of 1.51±0.84 (mean±standard deviation), which was strongly induced to 2.91 (±1.22) in breast cancers (*P*<0.001). Overall, 45 out of 55 tissue pairs (81.8%) showed this upregulation of FAK protein in tumours in comparison to normal tissue. Immunohistochemistry confirmed these findings with a significant higher score for tumours *vs* physiological tissue (1.0±0.63 *vs* 2.27±0.91; *P*=0.001). Interestingly, no overall significant difference in the mRNA levels (*P*=0.359) was observed. In conclusion, expression levels of the FAK protein are specifically upregulated in breast cancer in comparison to matched normal breast tissue supporting its pivotal role in neoplastic signal transduction and representing a potential marker for malignant transformation.

Neoplasia is characterised by abnormal cellular signalling due to an induction of protein tyrosine kinases and overexpression of other involved proteins like integrins (Hanahan and Weinberg, 2000; Hunter, 2000; Cruet-Hennequart *et al*, 2003). This phenomenon has implications for the initiation and progression of a variety of human cancers including gynaecological malignancies (Ullrich and Schlessinger, 1990; Hunter, 2000; Gabriel *et al*, 2002). Besides receptor tyrosine kinases, pure cytoplasmic tyrosine kinases are also involved in tumorigenesis and anchorage-independent growth of cells. An early and crucial event in signal transduction process in this regard is the phosphorylation of protein tyrosine residues. A pertinent downstream target is pp125 focal adhesion kinase (FAK), which binds to the *β*_1_ integrin subunit (Danker *et al*, 1998; Parsons, 2003). It represents a 125 kDa nonreceptor tyrosine kinase, which is ubiquitously expressed throughout development, widely detectable in adult tissues and specifically localised in focal adhesions, which represent contact sites for extracellular matrix interactions. Focal adhesion kinase holds a key position in the signal transduction network and is phosphorylated in response to clustering of integrins, cell spreading or formation of focal adhesions and acts as a scaffold for the assembly of multiprotein signalling complexes (Zachary and Rozengurt, 1992).

Functional FAK activities include cell cycle regulation and apoptosis (Xu *et al*, 1996; Zhao *et al*, 1998; Beviglia *et al*, 2003). Several studies confirmed a pivotal role of FAK in tumorigenesis and metastasis with elevated expression levels in various human malignancies (Owens *et al*, 1995; McCormack *et al*, 1997; Agochiya *et al*, 1999; Judson *et al*, 1999; Oktay *et al*, 2003). These findings were associated with enhanced cell migration (Cary *et al*, 1996) and proliferation (Wang *et al*, 2000). In lung cancer, the induced phosphorylation of pp125FAK was positively correlated to lymph node metastasis (Imaizumi *et al*, 1997). The tumour biological importance of FAK was confirmed by experiments targeting pp125FAK with antisense oligonucleotides leading to an inhibition of cell migration, invasion and proliferation as well as an induction of apoptosis and enhanced sensitivity to campothecins (Hungerford *et al*, 1996; Golubovskaya *et al*, 2002; Satoh *et al*, 2003).

So far there are only few, sometimes controversial reports linking FAK to breast cancer (Glukhova *et al*, 1995; Cance *et al*, 2000; Golubovskaya *et al*, 2002; Oktay *et al*, 2003; Satoh *et al*, 2003). Two studies found an upregulation of FAK expression during tumorigenesis from physiological tissue, via DCIS into invasive cancer (Cance *et al*, 2000; Oktay *et al*, 2003). Their results were compromised by the heterogeneity of tissue samples with benign specimen, DCIS and invasive tumours from different patients as well as low sample numbers. Two recently published large studies on 629 and 162 invasive breast cancers, respectively, found a correlation between FAK expression and an aggressive tumour phenotype. High FAK protein levels were associated with Her2/neu amplification and high tumour grading by immunohistochemistry, but both studies did not focus on the transition from normal to malignant breast tissue (Lark *et al*, 2005; Schmitz *et al*, 2005).

Our analysis focussed on the characterisation of pp125FAK expression in matched pairs of normal and malignant breast tissues from 55 individual patients on the mRNA as well as functional protein level.

## PATIENTS AND METHODS

### Patients and tissues

Fresh frozen tumour and physiological tissue samples of 55 consecutive patients who had surgery for primary breast cancer at the Department of Obstetrics and Gynaecology of Freiburg in 2003 were analysed. The patients had a mean age of 61 years. After approval by the local ethics committee (No. 313/2002) and written informed consent of each single patient, the removed tissue was sent immediately to the local pathologist for frozen section analysis. Tumour samples and corresponding physiological tissue of the same patient were frozen in liquid nitrogen and stored at −80°C until further analysis.

Two specialised and well-trained breast pathologists performed the characterisation of the tissue samples. The tumours were classified according to the TNM System. For histological analysis, tissue samples were fixed in 4% formalin and stained with haematoxylin–eosin. Additional immunohistochemical analysis was performed when necessary. The mean diameter of the tumours was 28.8±31.4 mm (mean±standard deviation (s.d.)). Histological type and tumour size were determined according to the WHO criteria (WHO, 1981). We found 37 (67.3%) ductal invasive, 13 (23.6%) lobular invasive and five (9.1%) other types of invasive breast cancers. Grading was assessed according to Elston and Ellis (1991). Axillary lymph node status was obtained by sentinel lymph node biopsy after radionuclid labelling or axillary dissection in case of metastatic sentinel lymph nodes. Oestrogen and progesterone receptor status was determined by immunohistochemistry. The DAKO Hercept Test™ (DakoCytomation, Glostrup, Denmark) was used to assess the HER2/neu status. Detailed clinical data of the patients are given in Table 1.

### Protein isolation and Western blot

Total cellular protein was isolated from the inter- and phenol phase from the initial homogenate after precipitation of the DNA (0.3 ml 100% of ethanol per 1 ml of TRIzol) as described previously. Briefly, proteins dissolved in the phenol–ethanol supernatant were precipitated with isopropyl alcohol (1.5 ml per 1 ml TRIzol), pelleted (12 000 × **g**, 4°C, 10 min) and washed three times with 0.3 M guanidine hydrochloride in 95% ethanol. Finally, the pellets were resuspended in 1% SDS solution and incubated at 50°C for complete dissolution. Insoluble material was removed by centrifugation (10 000 × **g**, 10 min, 4°C) and the supernatants were stored at −80°C until further analysis. For SDS–PAGE (10% gel), 20 *μ*g of total protein were applied per lane and separated. Proteins were electroblotted onto a PVDF membrane (PolyScreen, NEN Life Science, Boston, MA, USA) at 110 V for 1.0 h at 4°C. Unspecific binding sites of the membrane were blocked with 5% Blotto/PBST prior to incubation with the specific monoclonal FAK antibody (BD Biosciences, Franklin Lakes, USA) diluted 1 : 1250 in blocking solution. Bound primary antibody was visualised using horseradish-peroxidase labelled secondary goat-anti-mouse-IgG-antibody (Pierce, Rockford, USA) diluted 1 : 5000 in conjunction with a chemiluminescence detection system (Amersham Biosciences, Buckinghamshire, UK). Protein loads were normalised against *β*-actin and staining intensity was categorised by a visual score ranging from 0 to +5.

### Immunohistochemistry

Formalin-fixed, paraffin-embedded slides of 12 randomly chosen patients were soaked in Roticlear (Pure Science, Wellington, New Zealand) and rehydrated in a graded alcohol series (100–50%). To recover antigenicity we used the Antigen Retrieval System (BioGenex, San Ramon, USA) once for 60 min in an autoclave at 60°C. Afterwards, the slides were washed in PBS. The primary monoclonal antibody was specific for FAK (Clone No. 610087, BD Transduction Laboratories, Franklin Lakes, USA). Sections were incubated with the primary antibody diluted 1 : 500 in PBS for 60 min at 37°C. After rinsing in PBS sections were incubated for another 30 min with the link antibody, horseradish-peroxidase labelled secondary goat-anti-mouse-IgG-antibody (Pierce, Rockford, USA) diluted 1 : 500 in PBS. Afterwards the sections were coated with DAB staining reagent for 10 min at room temperature. Sections were rinsed in PBS and counterstained with haematoxylin, rinsed in water, dehydrated in 100% ethanol and equilibrated in xylol before mounting. The semiquantitative score ranged from negative to weak and strong staining reaction, assessing only the intensity (score 0 to +3).

### RNA isolation and RT–PCR

Tissues from 32 randomly chosen patients were minced on dry ice before treatment with a tissue homogeniser (Polytron, Littau, Switzerland) in Trizol solution (1 ml per 100 mg tissue, three times 10 s; Gibco-BRL, Gaithersburg, MD, USA). After 5 min of incubation at room temperature (RT), chloroform (0.2 ml per 1 ml of Trizol) was added, the solution was shaken vigorously and incubated (RT, 5 min). After centrifugation (12 000 × **g**, 15 min, 4°C) total RNA was precipitated by addition of isopropyl alcohol to the aqueous phase (0.5 ml per 1 ml of Trizol, 10 min RT). The RNA was pelleted by centrifugation (12 000 × **g**, 10 min, 4°C), washed with 75% ethanol (1 ml of ethanol per 1 ml of TRIzol reagent used for the initial homogenisation), recovered by centrifugation (7500 × **g**, 5 min, 4°C) and finally dissolved in RNAse-free water and stored at −80°C for further analysis.

In total, 5 *μ*g of RNA, as determined by optical densitometry, were used for cDNA synthesis using M-MuLV reverse transcriptase (Perkin-Elmer, Branchburg, NJ, USA) and oligo-dT primers. For amplification of cDNA, primers specific for FAK and 18S as control were used.

Primers were as follows:

FAK:

FAK S: 5′-TTA GAC AAT TTG CCA ACC TT-3′
FAK AS: 5′-CAC CTT CTT TCT GAG GTC TG-3′

18 S:

18S S: 5′-AAC TCA CTG AGG ATG AGG TG-3′
18S AS: 5′-CAG ACA AGG CCT ACA GAC TT-3′

Each tube contained 1 *μ*l of sense and antisense primer, respectively, dNTps, buffer and Taq polymerase in a total volume of 50 *μ*l. Amplification was for 30 cycles consisting of denaturing at 94°C (60 s), annealing at 55°C (30 s) and extension at 72°C (45 s).

For visualisation of amplified cDNA, 10 *μ*l from each reaction were submitted to electrophoresis on an agarose gel (2%). PeqGOLD DNA Sizer XI or XII (Peqlab Biotech, Erlangen, Germany) was used for size determination. Expected amplicon sizes were 417 bp for FAK and 305 bp for 18S, respectively.

### Statistical analysis

To allow a reliable statistical analysis in the given population, the results of the clinical and histological examination were dichotomised according to Table 1. The percentage of amplified FAK cDNA in correlation to the amplified 18S cDNA was used in the RT–PCR analysis. The *χ*^2^ test was applied to analyse the relation between the clinical and histological parameters and the test results. *T*-test for paired samples compared mean values of the tumour and normal tissue analysis. All *P*-values are two tailed. Any *P*-value lower than 0.05 was considered to be statistically significant. The analysis was performed with the SPSS Software package version 13.0.

## RESULTS

For this study, matched tissue pairs (*n*=55) of invasive breast cancer and corresponding physiological breast tissue of the same patient were analysed for their pp125FAK protein expression by Western blot analysis and immunohistochemistry as well as for RNA expression levels by RT–PCR.

### pp125 FAK protein concentration is increased in breast cancer

**Figure 1:**
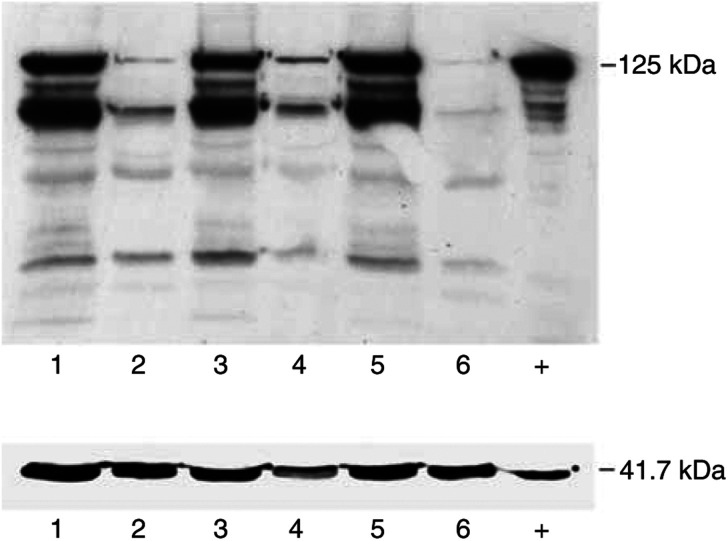
Induction of pp125 FAK expression in breast cancer. Matched pairs of normal and malignant tissue specimen from the same patient were analysed for their FAK expression by Western blot. Lane 1, tumour 1; lane 2, normal 1; lane 3, tumour 2; lane 4, normal 2; lane 5, tumour 3; lane 6, normal 3; +, positive control. pp 125 FAK corresponding to 125 kDa, *β*-actin corresponding to 41.7 kDa as internal control.

**Figure 2:**
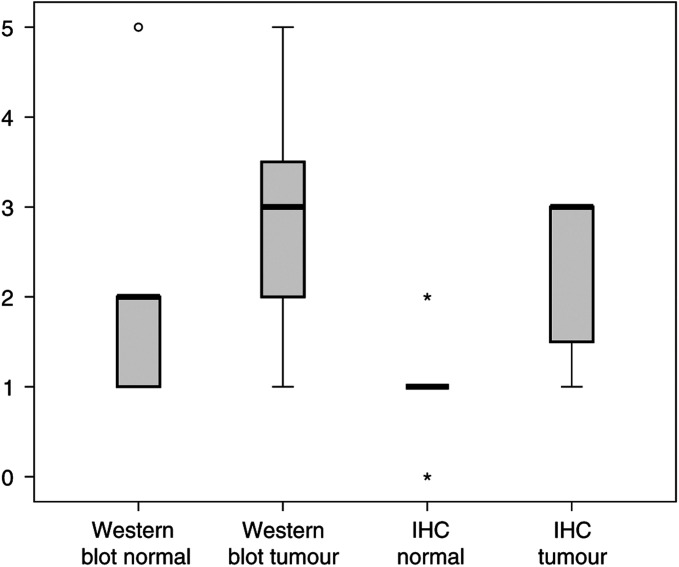
Box plot analysis of the Western blot score and immunohistochemistry of pp125 FAK expression in the breast. Matched pairs of normal and malignant breast tissue were analysed by Western blot with a specific monoclonal FAK antibody. The bold black line represents the median (50% percentile), the grey boxes show the 25–75% percentile, the thin lines under and above the grey boxes characterise the minimal and maximal values, the asterisks and circles represent rouge results.

To examine the expression profile of the pp125FAK on the functional level, we analysed at first the matched tissue samples by Western blot using the monoclonal antibody described above (Figure 1). The additional bands ranging from 70 to 85 kDa are known to be caspase cleavage products and were previously described (Gervais *et al*, 1998; van de Water *et al*, 1999; Mao *et al*, 2003) and do not compromise the value of the 125 kDa band. The mean score was 1.51±0.84 in physiological tissue *vs* 2.91±1.22 in tumours; this difference was highly significant (*P*<0.001) (Figure 2).

In more detail, the analysis of the score distribution revealed that 50 out of 55 normal tissue specimen (90.9%) were valued with a score of 0 to +2. Interestingly, score +1 was given in 30 out of 55 probes or 54.5% of all physiological tissue specimen. Only five (9.1%) samples were detected with a score of +3 or +5 (Table 2).

In contrast, invasive breast cancer displayed a strong FAK expression. Score +3 to +5 was found in 33 out of 55 (60%) specimen, with 17 out of 55 (30.9%) in the highest two score ranks +4 and +5. Only seven out of 55 tumours (12.7%) got a score of +1 (Table 2).

In a further step we analysed the relation between the histopathological and clinical parameters shown in Table 1 and the pp125 FAK protein expression. The *χ*^2^ test revealed only a significant positive correlation between pp125 FAK expression and high tumour grading (*P*=0.29), while all other parameters including the Her2/neu status showed no significant correlation.

### FAK protein expression is specifically upregulated in breast cancer

The immunohistochemical analysis of pp125 FAK expression in tumours *vs* corresponding and physiological breast tissue showed comparable results like the Western blot analysis.

**Figure 3:**
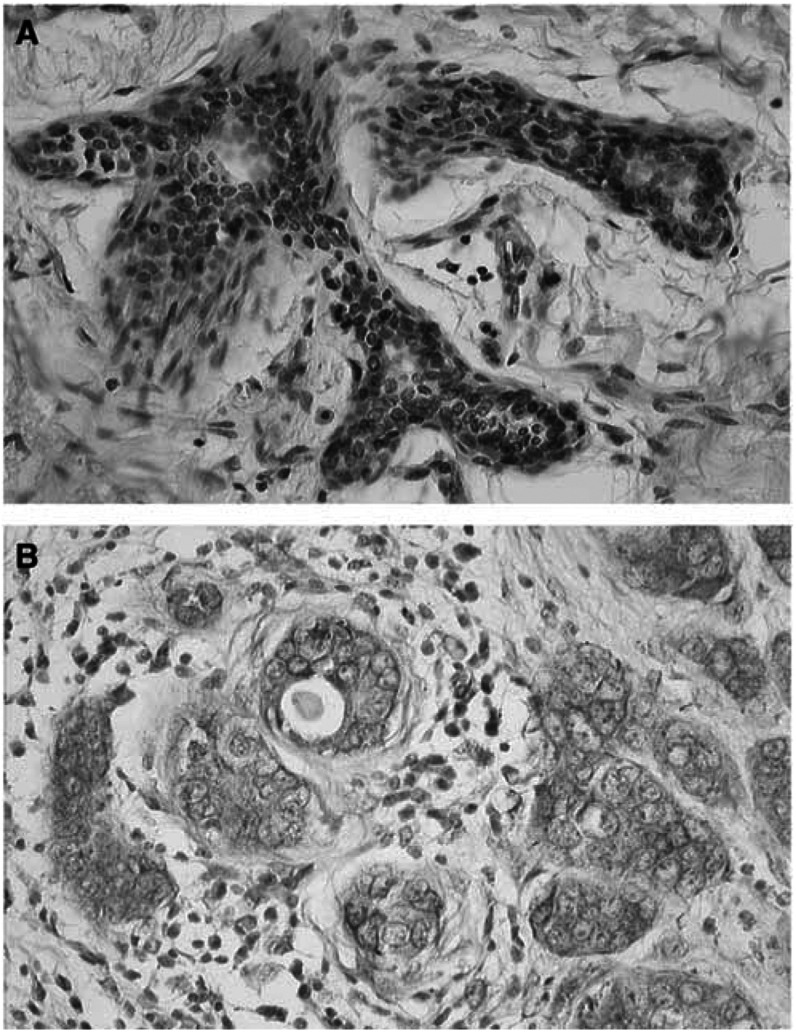
Induction of pp125 FAK expression in malignant *vs* normal breast tissue. Immunohistochemical analysis of pp125 FAK expression in a matched pair of physiological (**A**) and malignant (**B**) breast tissue of the same patient. While the physiological tissue reveals only a very weak to no expression of FAK, invasive breast carcinoma cells are characterised by a specific membranous expression of FAK (dark brown).

In the normal epithelial cells of the breast in general, a weak to no expression was observed with a mean score of 1.0±0.63. In invasive breast cancer we found a site-specific induction of pp125 FAK expression (mean score±s.d.: 2.27±0.91) (Figure 2). This difference was highly significant in the statistical analysis (*P*=0.001). The expression was mostly restricted to the membrane of the epithelial cancer cells, and sometimes also present in a few stromal cells (Figure 3A and B). In parallel to our Western blot results, the matched pair analysis revealed in all but one of examined tissue samples an induction of pp125 FAK expression in tumours *vs* their corresponding physiological control.

### FAK mRNA is inhomogeneously expressed in breast cancer

**Figure 4:**
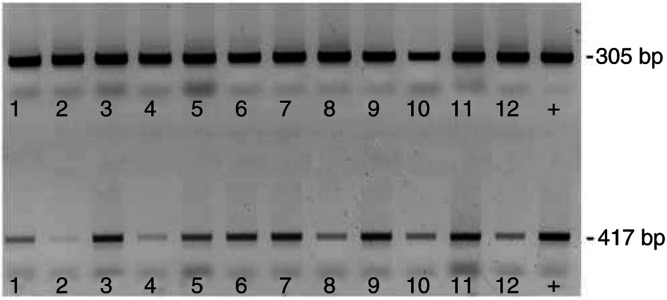
Induction of pp125 FAK RNA levels in malignant *vs* normal breast tissue. RT–PCR analysis of pp125 FAK RNA expression in matched pairs of normal and malignant breast tissue using FAK-specific primer (417 nbp) and for 18S ribosomal RNA as an internal control (305 bp). Lane 1, tumour 1; lane 2, normal 1; lane 3, tumour 2; lane 4, normal 2; lane 5, tumour 3; lane 6, normal 3; lane7, tumour 4; lane 8, normal 4; lane 9, tumour 5; lane 10, normal 5; lane 11, tumour 6; lane 12, normal 6; +, positive control.

Our RT–PCR analysis of matched tissue pairs revealed strikingly heterogeneous results for FAK mRNA levels in contrast to FAK protein. In some cases we found in the matched pair analysis a marked induction of FAK mRNA expression when normalised against 18S ribosomal RNA (Figure 4A). However, the overall statistical analysis could not confirm a general significant induction of FAK mRNA in the neoplastic lesions (*P*=0.359).

## DISCUSSION

Overexpression of tyrosine kinases is a frequently observed phenomenon in neoplastic cells. The central role of pp125FAK in signal transduction with potential implications in cell adhesion and migration suggests a pivotal role for this protein in solid tumours. Two just recently published studies found a strong correlation of FAK levels to an aggressive breast cancer phenotype esp. to grading and Her2/neu amplification (Lark *et al*, 2005; Schmitz *et al*, 2005).

Our results with a focus on a neoplastic transition from normal to malignant breast tissue support the hypothesis of an important functional role for FAK in breast cancer biology. The matched pair analysis of tissue specimen from the same patient revealed a gross induction of FAK in the majority of cases. Our Western blot analyses confirmed overexpression of pp125FAK functional protein in breast cancer tissue compared to matched normal controls. Low levels of pp125FAK protein were measured in physiological breast tissue, which is in accordance to experiments by Owens *et al* (1995), who found an elevated pp125FAK expression in 22 of 25 invasive and metastatic breast tumours compared to normal tissue. While speculating on possible reasons for these high levels, it should be considered that pp125FAK was shown to be an important survival signal for the anchorage-dependent growth of cells (Wang *et al*, 2000), and inhibition of pp125FAK resulted in consecutive cell death (Hungerford *et al*, 1996; Xu *et al*, 1996; Hauck *et al*, 2001; Golubovskaya *et al*, 2002). Our RT–PCR analysis revealed only slightly and statistically not significantly elevated FAK mRNA levels. This compromises the earlier postulated hypothesis of an upregulation of FAK mRNA and protein expression in breast cancer due to an increased copy number of the fak gene on chromosome 8q (Agochiya *et al*, 1999). The mRNA data of our study suggest that the protein induction is not based on primary transcriptional effects but most likely due to post-transcriptional or post-translational processing.

There are only few reports linking pp125FAK to breast cancer tumorigenesis (Glukhova *et al*, 1995; Cance *et al*, 2000; Golubovskaya *et al*, 2002; Oktay *et al*, 2003). Two comparative studies demonstrated an upregulation of pp125FAK expression in the transformational process from physiological tissue, via DCIS into invasive cancer (Cance *et al*, 2000; Oktay *et al*, 2003) using Northern or Western blotting techniques or immunohistochemistry. Cance *et al* (2000) found a moderate to strong expression in 14 out of 18 tumour samples and described a wide variability in the percentage of stained tumour cells ranging from 5 to 100%. Furthermore, the examined breast cancer specimens represented a variety of cell types, including pure invasive ductal, mixed ductal and lobular, and pure invasive lobular carcinomas. The authors interpreted pp125FAK overexpresion as an early event in tumorigenesis observed already in the state of DCIS (Cance *et al*, 2000). These results were confirmed by Oktay *et al* (2003), who detected FAK overexpression already in atypical ductal hyperplasia and 100% of the examined DCIS.

However, the significance of these findings remained unclear, especially since Glukhova *et al* (1995) could not demonstrate a pp125FAK overexpression in human breast cancer at all.

In our experiments we used total tumour tissue homogenates and breast cancer samples which were not enriched as epithelium pp125FAK from surrounding stromal contaminants must be taken into consideration. Thus, we cannot conclude that pp125FAK is necessarily upregulated exclusively in the epithelial fraction. However, our immunohistochemical data confirmed a specific FAK overexpression in tumour cells with only minor presence in surrounding tissue. These findings support the hypothesis of a specific upregulation in tumour cells.

The tumour biological importance of pp125 FAK overexpression in breast cancer is still not clear. An interesting link might exist to Her2/neu, since Vadlamudi *et al* (2003) suggested that HER2 signalling events influence metastasis of breast cancer cells through a signalling pathway involving phosphorylation of pp125FAKs tyrosine 861 via activation of Src. These results were supported by the studies of Schmitz *et al* (2005) and Lark *et al* (2005) both who found a strong correlation to the Her2/neu status and grading. We could not confirm the Her2/neu results in our study population but found in accordance to Schwartz and Schmitz *et al*, the strong correlation of FAK expression and tumour grading. These data support the evidence for an association of FAK with a more aggressive tumour phenotype.

In summary, the frequent and specific upregulation of pp125FAK in human breast cancer suggests an important functional role in neoplastic signal transduction and represents a potential marker for malignant transformation in breast epithelium and target for therapeutic interventions.

## Figures and Tables

**Table 1:** Clinical data of 55 breast cancer patients

	**No (%)**	**Yes (%)**
Age ⩽50 years	13 (23.6)	42 (76.4)
Tumour diameter <20 mm	29 (52.7)	26 (47.3)
Grading 1and 2	46 (83.6)	9 (16.4)
Her 2/neu score 3+	46 (83.6)	9 (16.4)
Lymph node metastasis	37 (67.3)	18 (32.7)
Distant metastasis	49 (89.1)	6 (10.9)
Multilocal growth pattern	30 (54.5)	25 (45.5)
Lymphangiosis	39 (70.9)	16 (29.1)
Oestrogen receptor	15 (27.3)	40 (72.7)
Progesterone receptor	19 (34.5)	36 (65.5)

**Table 2:** Score distribution of FAK protein expression in breast tissue

	**Normal tissue**		**Tumour tissue**	
**Score**	***n***	**%**	***n***	**%**
0	2	3.6	0	0
1	30	54.5	7	12.7
2	18	32.7	15	27.3
3	4	7.3	16	29.1
4	0	0	10	18.2
5	1	1.8	7	12.7
Total	55	100.0	55	100.0

Western blot score of pp125 FAK protein expression ranged from 0 to +5.
